# Themes arising in clinical consultation for therapists implementing family-based treatment for adolescents with anorexia nervosa: a qualitative study

**DOI:** 10.1186/s40337-017-0161-3

**Published:** 2017-09-04

**Authors:** J. Couturier, J. Lock, M. Kimber, G. McVey, M. Barwick, A. Niccols, C. Webb, S. Findlay, T. Woodford

**Affiliations:** 1 Department of Psychiatry and Behavioural Neurosciences, McMaster University, 1200 Main St. West, Hamilton, ON L8N3Z5 Canada; 2 Department of Pediatrics, McMaster University, Hamilton, Canada; 3 Department of Clinical Epidemiology and Biostatistics, McMaster University, Hamilton, Canada; 4 Department of Psychiatry & Neurosciences, Stanford University, Stanford, USA; 5 Offord Centre, McMaster University, Hamilton, Canada; 6 Toronto General Hospital Research Institute, University Health Network, Toronto, Canada; 7 Dalla Lana School of Public Health, University of Toronto, Toronto, Canada; 8 Department of Psychiatry, University of Toronto, Toronto, Canada; 9 Research Institute, The Hospital for Sick Children, Toronto, Canada

**Keywords:** Implementation, Family-based, Adolescent, Eating disorders

## Abstract

**Background:**

Our study aims to explore and describe themes arising in sessions of clinical consultation with therapists implementing Family-Based Treatment (FBT) for adolescents with Anorexia Nervosa (AN). There is currently no literature describing the content of clinical consultation for FBT. Thus, this knowledge will add to the evidence-base on what therapists need from consultants in ongoing clinical consultation.

**Methods:**

Eight therapists at four sites participated in this study, which spanned a two-year period. Following a two-day training workshop, each therapist treated at least one adolescent patient presenting with a restrictive eating disorder with FBT, focusing on adherence to the treatment manual. Clinical consultation sessions occurred monthly and were led by an external FBT expert. Thirty-five (average per site = 9) audio recorded group clinical consultation sessions were transcribed verbatim and coded for themes. Twenty percent of the transcripts were double-coded to ensure consistency. Fundamental qualitative description guided the sampling and data collection.

**Results:**

Thematic content analysis revealed ten common themes relating to the provision of clinical consultation to therapists implementing FBT in clinical practice: encouraging parental meal time supervision,discussing the role of mothers, how to align parents, ensuring parental buy-in, when to transition to Phase 2, weighing the patient and the patients’ knowledge of their weight, the role of siblings in FBT sessions, how best to manage patient co-morbidities, the role of the father in FBT and how best to manage the family meal.

**Conclusions:**

In conclusion, clinical consultation themes aligned with many of the central tenets of FBT, including how to help parents align their supportive approach during the refeeding process, and how to help parents assume control of eating disordered behaviours. This knowledge helps to guide consultants to anticipate common issues brought forward by therapists attempting to implement FBT.

## Plain English Summary

This study examined themes arising during clinical consultation by phone for therapists attempting to apply Family-Based Treatment for adolescents with eating disorders. Therapists were attempting to follow a manual describing this type of psychotherapy and were seeking guidance from an expert in Family-Based Treatment. This study showed that therapists asked for help around how to encourage parental supervision of their child’s eating, as well as how to help parents stay aligned and “on the same page” during this therapy. This study indicates that therapists asked for guidance repeatedly with respect to issues consistent with the Family-Based Treatment manual.

## Background

Family-Based Treatment (FBT) has emerged as a first line treatment for children and adolescents with eating disorders due to its superior impact on outcomes [1–3]. A previous study involving 40 therapists in Ontario, Canada, identified a number of challenges and facilitating factors to the uptake of FBT in clinical practice [4, 5]. These challenges included the perception that FBT does not address comorbid conditions, that there are difficulties engaging parents and other members of the medical team, and that the therapists lack sufficient training and supervision to successfully implement the FBT model with high fidelity [4, 5]. The majority of therapists and administrators identified comprehensive training by a local and/or international FBT expert as essential for successful adoption, with 38 of the 40 therapists (95%) requesting a province-wide training initiative that would involve didactic training and ongoing clinical consultation. All of the therapists indicated that support from administrators was necessary for training and for practice change to occur. In addition, they emphasized that support from team members, especially physicians, was crucial. Nineteen of the 40 therapists (46.5%) reported having previously received FBT training, with 31 of the 40 (77.5%) reporting they had read some or the entire FBT manual [6]. Despite these educational efforts, however, none of the therapists practiced the model with high fidelity [4]. All participants in a similar study of program administrators (*n* = 11) reported that every therapist in their program required further training *and* ongoing supervision to deliver FBT with fidelity [7].

When evidence-based treatments are not provided with fidelity, outcomes are less likely to approximate those found in research trials [8]. Therefore, ongoing training and clinical consultation support serve to develop and sustain therapist competence. Many terms have been used to describe this ongoing contact for educational purposes, including clinical consultation, supervision, coaching and ongoing support [9]. Generally, supervision is provided by a lead clinician/manager who is a member of the service provider organization, whereas clinical consultation (or coaching) implies external support provided within dissemination and implementation efforts [10]. The current study uses the term *clinical consultation*, as external support was provided for therapists implementing FBT within their organizations.

There is evidence that ongoing clinical consultation can increase fidelity to an evidence-based treatment (EBT) compared to provision of a didactic training workshop only [11]. Schwalbe and colleagues [11] completed a meta-analysis looking at Motivational Interviewing (MI) implementation with and without ongoing clinical consultation and found that those who did not receive ongoing clinical consultation showed diminished MI skills over a 6 month period, whereas, those who had ongoing consultation made skill gains over the same time period. The type of clinical consultation provided may also be significant (e.g. [12]). Although there is scant literature on this topic, Bearman and colleagues [8] randomized therapists to two groups receiving different components of supervision following a 3 h workshop on providing cognitive therapy. Therapists in both the intervention and “supervision as usual” arms received supervision sessions that covered rapport building, agenda-setting, case discussion and conceptualization, treatment planning, developing therapeutic alliance, and case management strategies. The intervention arm also received feedback on their practice based on reviews of audio recordings, as well as technique modeling, and role-playing. Findings revealed that therapists randomized to the intervention arm continued to improve in knowledge, attitudes, and fidelity over the three-week period. Participants in the supervision as usual group did not make further improvements in their fidelity to the treatment model following the initial training workshop [8]. A number of review papers have highlighted that ongoing clinical consultation is critical to promote uptake and adherence to EBTs [13, 14]. Furthermore, there is tremendous need for research to identify effective clinical consultation strategies and the mechanisms through which clinical consultation impacts implementation outcomes [13–16].

Clinical consultation practices for FBT have been previously described on a superficial level in randomized trials within research settings [3, 17, 18]. These practices typically included a training workshop followed by weekly supervision provided by an FBT expert either in person or over the phone, and included information based on reviews of audio or video recordings of therapists providing FBT [3, 17, 18]. FBT implementation studies have also been completed in academic centers different from those of the primary research on FBT, using a workshop and weekly supervision by a local expert [19–21]. However, no studies have focused on the content of FBT clinical consultation sessions. Thus, the purpose of this study was to explore and describe themes arising in clinical consultation of therapists implementing FBT for adolescents with eating disorders, with the hopes that this knowledge could help consultants to anticipate themes which may arise in their own work and will add to the evidence-base on what therapists need from consultants in ongoing clinical consultation within this treatment model.

## Methods

### Design

Data for this study come from a larger, multi-site (*n* = 4), mixed method, pre-post implementation study that sought to use evidence-based models of implementation science to broaden the capacity for Ontario-based therapists to deliver FBT to children and adolescents diagnosed with eating disorders. As such, the context for FBT implementation was research-initiated and supported, the implications of which we discuss later relative to sustainability. Briefly, informed by the National Implementation Research Networks Active Implementation Framework [22] and the Consolidated Framework for Implementation Research [23, 24], the larger study purposefully recruited therapists, physicians and administrators in four Ontario-based pediatric eating disorder programs to undergo training and clinical consultation in the FBT model, and explored implementation processes and therapist experiences of clinical consultation. To work with the research team and to support the sustainability to FBT at each of their respective programs, each of the participating organizations was asked to identify an implementation team that consisted of an administrator/manager, a lead therapist and a physician who would be charged with supporting FBT training, supervision, implementation and research processes for this study. In addition, the implementation team was asked to identify therapists in their program who were most appropriate and willing to undergo training in the FBT model, receive clinical consultation with respect to their FBT practice and willing to participate in the study’s research processes. Although the administrators and physicians did not participate in the clinical consultation reported on in this study, they did participate in monthly implementation calls that will be described in a forthcoming paper. We did not limit the number of therapists who could participate in the FBT training and implementation endeavour, but implementation teams were limited to nominating therapists who would ultimately deliver FBT to children and adolescents diagnosed with Anorexia Nervosa (AN) or Bulimia Nervosa (BN) over the entire course of the study.

### The treatment model

FBT is an outpatient, intensive treatment in which the family is the primary resource to re-nourish the affected child [6]. FBT involves three phases of treatment over 9 to 12 months. The first phase focuses on helping the family to restore the child’s weight and interrupt disordered eating behavior. The second phase involves transition of control of eating behavior back to the adolescent. The third and final phase addresses developmental issues such as physical development, peers and dating, and separation and individuation. FBT requires the therapist to weigh the patient, graph the weight and reveal the weight to the patient and family at each session. The therapist coaches the parent(s), who are charged with making decisions about nutrition and exercise without consultation from a dietician [6].

### Study procedures and data collection

The administrator, lead therapist, physician and selected therapists from each participating organization participated in a two-day FBT training workshop provided by an international FBT expert (JL) and that was organized and attended by some members of the research team (JC, MK, TW, SF, CW). Following the training workshop, all lead and selected therapists (*n* = 8) were asked to treat at least one adolescent patient with AN or BN using the FBT model, and participate in monthly telephone clinical consultation (1 h per call). Adolescent patients and their families provided consent to be included in the study. FBT sessions with consenting adolescents and their families were audio recorded, shared with the research team through an online encrypted system, and reviewed for fidelity to the FBT model by the consultant (JC). The consultant is a certified FBT therapist who has received extensive training by an international FBT expert (JL). Implementation issues were captured during separate monthly phone calls with the implementation teams and will be reported on elsewhere. Clinical consultation recordings were transcribed verbatim for qualitative data analysis, differentiating the comments of the therapists and the consultant. The audio recording and transcription of clinical consultation calls were guided by the principles of fundamental qualitative description [25]; and more specifically, by the desire to provide a methodologically rigorous description of FBT clinical consultation. In contrary to other forms of qualitative methodology—like ethnography, grounded theory, phenomenology or interpretive descriptive, analysis within fundamental qualitative description stays close to the data and is particularly relevant for understudied research areas to depict the reality of experiences. That is, it does not involve interpretation within the analytical framework, but rather, seeks to generate a comprehensive picture of an experience so as to advance the field toward more interpretive questions [26].

This study received ethical approval from the Hamilton Health Sciences/McMaster Faculty of Health Sciences Research Ethics Board as well as the ethics boards at all participating sites.

### Data analysis

Conventional content analysis [27] was used to develop an analytical codebook to guide first and second levels of coding. Conventional content analysis does not assume any underlying explanation or explanatory framework for participants’ experiences; and in this manner, allows for themes generated through qualitative data analysis to inductively emerge from within and across the collected data. Content analysis is particularly suited to fundamental qualitative description methods given that data analysis is intrinsically linked to what is actually said by participants, provides the basis of recommendations generated from the research, and can provide a clear picture for moving the field forward with respect to clinical consultation practices within implementation research, more broadly.

Each transcript was read in its entirety and line-by-line coding was conducted to identify key concepts emerging from the transcripts. A codebook with definitions of each code and theme was generated and refined through multiple readings of the transcripts, in consultation with the research team, as well as through the process of theoretical memoing [28]. Memoing is an analytic process that aids in the development of ideas about codes and themes, and their interaction with one another [28]. To ensure dependability of data analysis, all clinical consultation call transcripts were coded by TW, an experienced qualitative data coder and 20% of these transcripts were independently double-coded by the principal investigator (JC). Disagreements in the development and application of particular codes were resolved through consensus-making discussions with a third team member (MK). Specifically, the initial definition of the code or theme was reviewed and references to that code or theme were discussed in relation to the initial definition until consensus was reached for the application of the code. As new codes and themes emerged from the transcripts, they were added to the codebook and documented in the study’s research audit trail. The collapsing of codes into categories was documented in the same fashion. Then, utilizing the constant comparative technique [29] codes were applied to the raw data and quotes were pulled from the data to reflect the coded perceptions expressed by the therapists participating in this study. Subsequently, summative content analysis was used to provide counts of codes within and across transcripts and organizations [27]. Data collection and analysis was dictated by the case-study nature of the present study, with analysis occurring until no new themes were emerging from the data that would alter the interpretation of the results. All coding was completed in Nvivo 10 (QSR International Pty Ltd., Version 8, 2008).

## Results

### Descriptive data

Eight therapists (six female, two male) participated in this study from four different sites. Four therapists were social workers, two were psychologists, and two were psychiatrists. Therapists ranged in age from 28 to 60 years and had worked in eating disorder services for an average of 7.4 years (range 1 month to 14 years). The number of clinical consultation sessions provided was 35 with an average of nine sessions per site. The time period for the clinical consultation was 11 months (range 7 to 15 months). The number of clinical consultation sessions per therapist was 7.6 (range 5 to 11). All clinical consultation sessions were captured and analysed. In other words, there were no missed sessions or missing data.

Themes presented below are the ten most frequently occurring themes according to source in order of highest to lowest frequency.

### Clinical consultation themes

Although therapists were invited to enroll young people presenting with AN or BN, all patients involved in the study met criteria for AN as reported by the therapists. There were a number of overarching experiential themes emerging from this work, the first of which relates to how to support and encourage ***parental supervision of re-nourishment:*** The clinical consultation discussion on this theme was the most common one identified in this study (Table 1). The theme captured how best to help parents be more effective in mealtime supervision, how to assist parents in understanding when they or their child was actually making good nutritional decisions, and how parents could navigate taking a leave of absence from work in order to attend to their ill child. In addition, therapists also asked for guidance about how to help parents to decide whether and how to delegate mealtime monitoring to a teacher or other school personnel once the child returned to school. Therapists needed to be encouraged to obtain more details surrounding mealtime activities and decisions:

*Consultant: Are you reviewing what the meals and snacks consist of?*

*Therapist: Not in detail, no. Not in detail like “Tell me what is in each meal?” Stuff like that. I am reviewing that she is having a meal, is a parent there, and you know is it enough food that you know she’s going to gain weight.*

*Consultant: It might help to get a little more detail, because since she is stalling a bit (with weight gain), to try to get a bit more detail and help them try to figure out what they are going to add to increase.*

**Table 1:** Themes arranged in order of frequency of occurance within sources along with numberreferences to each theme

Theme	Sources (*n* = 35)	References
Parental supervision	29	130
Assisting Mother	26	93
Alignment of Parents	24	78
Parental Commitment	22	76
Phase 2 Transition	17	66
Weight	21	62
Siblings	20	52
Comorbidities	16	49
Involving Father	17	48
Family Meal	13	48

Another common clinical consultation theme was helping therapists understand how to **assist mothers** in the context of FBT. Although fathers were also involved in the study and attended appointments, issues on how to assist with mothers were more frequently raised, perhaps as mothers were more frequently the primary caregivers. This theme encompassed therapists’ concerns about how to manage maternal emotions, including anger and guilt. Mothers’ ability to balance being firm and demonstrating empathy toward their ill child were both important. In addition, therapists discussed the maternal history of an eating disorder as a possible barrier to helping the child eat enough to gain weight. Maternal anxiety about confronting the eating disorder, along with the associated distress of refeeding, was a common theme, illustrated here:

*Therapist: …it has been stated by mom that she doesn’t want to upset her daughter. She doesn’t want to challenge the eating disorder, she doesn’t want to make her angry, or stressed or upset, and as a result I have been fascinated that the daughter just sits there and says “but mom, I’m hungry”*

Therapists had to be encouraged to help parents separate the disorder from the person so that they did not feel they were upsetting their daughter, but were rather addressing the disease.

Therapists also discussed their difficulties in knowing how best to ***align parents***. Specifically, clinical consultation support was sought to help parents *agree* on how and what they wanted to do to help their child gain weight or to stop binge eating or purging. Guidance on how best to align parents’ perspectives, attitudes, and behaviours was an FBT clinical consultation theme regardless of the marital status of the parents. Some therapists mentioned that parents were critical of each other in front of the child, creating a split and making it more difficult for the parents to be successful in disrupting behaviors that maintained the eating disorder. In the following example, the therapist discusses how parents can work together when neither one really wants to be aligned with the other:

*Therapist: So I found it very difficult to get parents to work together as a team because Mom keeps saying, she feels, she doesn’t, like her dad [the husband] doing it. He’s no good at it. He can’t do it I don’t want him there. She doesn’t want him there, he never has been there, you know?*

In this situation the consultant encouraged the therapist to help parents work together and share in the workload in defeating AN.

***Parental Commitment in FBT*** was also mentioned frequently by therapists. Lack of maternal or paternal attendance at sessions was perceived by the therapist as a lack of commitment to FBT. Additionally, therapists voiced concern that parents often requested other forms of therapy for their child, including individual treatment or inpatient treatment; and this was despite having been made aware of the evidence supporting FBT effectiveness. Therapists also sought clinical consultation guidance when parents disagreed with weight targets for their child:

*Therapist: Like they’re saying “she’s cured and she’s at her healthy weight and when her weight was lower and Dr. X checked her estrogen levels and it was 300 and something and she’s always been teeny-tiny and skinny. Why are you still trying to put weight on her? We would just like to go and just keep her eating.”*

Therapists commonly sought guidance for supporting the ***transition to Phase 2 of FBT***, which involves allowing the adolescent more independence around eating. Clinical consultation discussions revolved around how to help parents prevent the adolescent from gaining control over these behaviors prematurely. Other subthemes related to the transition of the family to phase 2 of the FBT model included therapists needing help with identifying the signs indicative of an appropriate transition to Phase 2, including: weight gain, lack of resistance to eating foods that would cause anxiety in the past, and the return of menstruation (although the return of menses is not a requirement for phase 2 in the treatment manual). For example, one therapist discussed the case of an adolescent who was given control over food variety too quickly:

*Therapist: So her parents have backed off too much on the variety front as soon as her weight was restored.*

In this case, the therapist had already recognized the transfer of control had been executed too quickly and she pointed this out to the parents.

Several themes were less common, although important, for FBT clinical consultation, including: issues surrounding ***weighing the patient and the patients’ knowledge of their weight***, the role of ***siblings*** in FBT sessions, how best to manage patient ***co-morbidities***, the ***role of the father*** in FBT and, how best to manage the ***family meal***. Therapists often discussed the pros and cons of ***weighing*** the patient and communicating that information back to the patient and family, with the largest problem raised by the consultant that if the patient does not know his/her own weight, he/she cannot manage his or her own weight.

The role of ***siblings*** in FBT also emerged as a theme in clinical consultation discussions. Therapists shared that some siblings did not want to be involved in treatment, particularly as therapy progressed. Sometimes the patient did not want his or her siblings involved due to feelings of shame. In this case, the consultant encouraged the therapist to continue to advocate for sibling attendance and involvement as a strategy to support the ill child. Patient ***comorbidities*** were discussed along with physical violence in the home. Therapists discussed parental violence toward the child, but also violent behavior in the child directed toward the parent. In these instances, the consultant reviewed when it was important to consider calling child protective services, and when referral for psychiatric consultation for medication and/or admission might be helpful in circumstances of extreme agitation.

The role of the patient’s ***father*** in FBT was also a focus of clinical consultation discussions, with some therapists reporting that fathers voiced a fear of their own anger. In these cases, therapists were encouraged to help fathers externalize their anger toward the eating disorder and away from their child, which would help support their ability to actively participate in the refeeding process. Many therapists also discussed how to encourage father to attend the sessions and therapists were encouraged to come up with creative solutions, such as telephone involvement in the session by the father.

The family meal is one of the central components of FBT. Therapists observe a meal that parents bring in and therapists are to coach the parents to help their child eat one more bite. Therapists discussed the structure and flow of the ***family meal*** **,** reviewed its goals and tried to anticipate challenges. For example, one therapist who had never conducted a family meal looked for support around the potentially contentious nature of meal time and when to terminate the meal if the experience was getting too conflictual. In this situation, the consultant reviewed that the most important aspect to the family meal is not really whether or not the child has “one more bite”, but rather, how the family practices strategies to help reduce eating disordered behaviours.

## Discussion

This study is the first to take a qualitative approach to understanding the themes arising in clinical consultation for therapists implementing FBT in practice. Some themes were specific to the FBT model, including managing the family meal, parents assuming meal-time supervision, uniting parents, and weighing the patient and communicating the patient’s weight. These themes link to FBT components that have been associated with better outcomes [30]. Ellison and colleagues found that when parents took control, were united, externalized the illness and did not criticize the patient, better weight gain was predicted [30]. Although our study did not look specifically at patient outcomes, it is encouraging to know that the issues discussed in FBT clinical consultation related to salient aspects of FBT. Clinical consultation themes also aligned with findings from our previous qualitative research in which therapists identified several perceived barriers to FBT implementation, including weighing the patient, the management of co-morbidities, and how best to involve siblings in therapy [4, 5].

Therapists were keen to discuss the fundamental tenets of FBT – parents being in control of eating disorder behaviours and re-nourishment of their child, aligning of parental perspectives, attitudes and behaviours, and externalizing the illness – all of which were discussed in the didactic training workshop prior to the initiation of their clinical cases. Clinical consultation provided an ongoing forum for review and support around these key issues. This finding resonates with other research indicating that mental health therapists desire ongoing clinical consultation [8, 11]. The amount of clinical consultation provided may also be important, as demonstrated by Beidas and colleagues [31] who showed that therapist adherence and skill were related to the number of clinical consultation hours following a training workshop. Participants in their study received weekly clinical consultation for a three-month period while learning CBT for youth with anxiety. Each hour of clinical consultation improved adherence by .4 and skill by .3 on a 7 point Likert scale, suggesting a good return on investment [31]. How well the consultant fulfills their role is also important. A study examining consultant adherence to a clinical consultation protocol for multi-systemic therapy found a link between consultant adherence and therapist fidelity, as well as post-treatment youth behavior problems and functioning [32]. Specifically, therapist-perceived consultant expertise was positively associated with therapist adherence and improved youth outcomes [32].

In our FBT study, therapists received monthly clinical consultation for an average period of eleven months. This is in contrast to studies examining treatment outcome, which generally involves weekly supervision. Our study encouraged therapists to meet “in-house” on a weekly basis to discuss their cases in peer supervision, while providing clinical consultation on a monthly basis to support the implementation. It is quite possible that other themes arose within the in-house supervision and were not captured. In previous research, therapists have reported that effective elements of clinical consultation include connectedness with other therapists and the consultant, authentic interactions around clinical cases, and consultant responsiveness to therapist needs [33]. Although these clinical consultation elements were not explored in the current study, they would be important to explore in future FBT clinical consultation and implementation studies.

Interestingly, our findings indicate that the most common themes are all related to FBT content rather than process. Therapists did not discuss their level of comfort/discomfort or any confidence-related material. Perhaps this was because all of the participants were seasoned clinicians. If consulting to a more junior group of therapists, perhaps different issues might have emerged, particularly related to confidence, skill, or questioning the central tenets of FBT on a philosophical level. As clinical consultation was provided in a group format, it is also possible that therapists were reluctant to discuss issues of confidence or competence in front of their colleagues. There was also not as much focus on session process as opposed to session content. Again, therapists did not raise these issues frequently. Perhaps because FBT is quite a behavioural treatment with a well-described manual, therapists focused on the content. Potentially if the treatment were more of a psychodynamic model, themes related to process would have been more frequently discussed. In addition, it is quite possible that some of this process related material arose during in-house supervision, which was not captured in our data.

The limitations of our study also include the small sample size of eight therapists from four sites. Although we had representation from small and large health care centres, and our sample represented a range of therapist experience, it is probable that several contextual factors may have impacted their practice change (e.g., see [24]), and these were not captured in our study. In addition, therapists were each encouraged to take on two clinical cases to increase the clinical variety of families presenting for care, yet not all were able to recruit two families within the study period due to systemic barriers.

Although the current findings do not examine treatment fidelity and how this may relate to the clinical consultation model, we examine this aspect of our implementation model in a forthcoming paper. Sustainability of FBT implementation in these settings also needs to be addressed. Since FBT implementation was enabled through the support of a research grant, the future sustainability of manualized FBT could be jeopardized. Each site invited their lead therapist to provide regular, ongoing local supervision outside of the clinical consultation calls, and future data collection will inform as to whether this actually occurred. It is worth mentioning that the feasibility and therapist buy-in were largely due to the fact that the participating programs belonged to an established community of practice (website www.ocoped.ca). Our province is unique in that there are 22 programs situated within an established, coordinated and collaborative network of specialized eating disorder treatment services [34]. This demonstrates how networks such as this one provide a context from which we can examine implementation strategies, especially with respect to fostering uptake and sustainability of practice change. There is urgency to study and understand the variables important to successful community uptake of evidence-based practices given the seriousness of eating disorders in youth as well as the costs of treatment, where early and effective treatment can save lives and/or change the trajectory of chronic and debilitating illnesses. This study represents one of a few implementation research studies conducted within the field of eating disorder treatment and, aligns with current government efforts to bring implementation science to the field of mental health and addictions.

## Conclusions

Clinical consultation themes aligned with many of the central tenets of FBT, including how to help parents align their supportive approach during the refeeding process, and how to help parents assume control of eating disordered behaviours. This study demonstrates the most frequently arising themes in clinical consultation of those implementing FBT and provides guidance to potential consultants who can now anticipate the issues which will arise in their own consultation practice. It also provides valuable information to therapists implementing FBT with respect to common dilemmas faced by FBT therapists and some potential responses by a FBT consultant.

## Abbreviations

AN: Anorexia nervosa
BN: Bulimia nervosa
FBT: Family-based treatment
